# Diagnostic accuracy of 14‐3‐3 η protein in rheumatoid arthritis: A meta‐analysis

**DOI:** 10.1111/1756-185X.13921

**Published:** 2020-09-10

**Authors:** Decai Wang, Yalan Cui, Huiren Lei, Ding Cao, Guoting Tang, Haiming Huang, Ting Yuan, Lizong Rao, Biwen Mo

**Affiliations:** ^1^ Department of Respiratory and Critical Care Medicine Guangxi Zhuang Autonomous Region Education Department Key Laboratory of Respiratory Diseases Affiliated Hospital of Guilin Medical University Guilin China; ^2^ Department of Anatomy Guilin Medical University Guilin China

**Keywords:** diagnostic, meta‐analysis, rheumatoid arthritis, 14‐3‐3η (14‐3‐3 eta) protein

## Abstract

**Aim:**

To evaluate the overall diagnostic performance of 14‐3‐3 η protein in patients with rheumatoid arthritis (RA).

**Methods:**

PubMed, EMBASE, and Web of Science were searched to acquire eligible studies. Articles published in English before 20 February 2020 were included. Quality Assessment of Diagnostic Accuracy Studies 2 was used to evaluate the risk of bias and application concern of the included articles. Pooled analysis of diagnostic indicators of 14‐3‐3 η protein for RA was conducted by using a random effects model. Subgroup analysis was used to explore the sources of heterogeneity. Deeks' funnel plot asymmetry test was used to evaluate for the presence of publication bias.

**Results:**

A total of 13 studies (1554 positive and 1934 negative participants) were included. The pooled sensitivity and specificity were 0.73 (95% CI 0.71‐0.75) and 0.88 (95% CI 0.87‐0.90), respectively. The pooled positive/negative likelihood were 5.98 (95% CI 4.39‐8.14) and 0.28 (95% CI 0.21‐0.37), respectively. In addition, the pooled diagnostic odds ratio was 23.48 (95% CI 13.76‐40.08) and the area under curve was 0.9245. The results of subgroup analysis indicated that ethnicity and control group might be the source of heterogeneity. The results of sensitivity analysis were stable. No significant publication bias was found.

**Conclusions:**

The current evidence indicated that 14‐3‐3 η protein has moderate accuracy for the diagnosis of RA.

## 1 INTRODUCTION

Rheumatoid arthritis (RA) is a chronic, inflammatory joint disease of autoimmune nature, characterized by painful, swollen joints that can severely impair physical function and quality of life.^1^, ^2^ The disease affects women 2‐3 times more often than men and occurs at any age.^3^ The peak incidence is between ages 50‐60 years. In Western countries, the prevalence of RA is in the range of 0.5%‐1.0% in White individuals, while the prevalence ratios were 0.45, 0.69 and 1.02 for women of Hispanic, Asian or African‐American descent, respectively.^2^ RA is a heterogeneous disease, with variable clinical presentation and pathogenetic mechanisms involved between individuals with the same formal diagnosis or across different disease stages. The most prominent feature is symmetrical pain and swelling of the hands, wrists, feet, and knees (polyarthritis), although other joints may be affected. Some patients with RA may present or later develop disease manifestations in other organs, such as interstitial lung disease, pericarditis, pleural effusion, or bronchiectasis.^1^ RA is a major global public health challenge. The age‐standardized prevalence and incidence rates are increasing, especially in countries such as Canada, Paraguay and Guatemala.^4^ Thus, early identification and treatment of RA is vital, especially among females, in order to reduce the ongoing burden of this condition. In the early‐stage disease, patients with RA who were treated with early remission induction therapy retain normal function, and they almost had no clinically relevant joint injuries. And the sooner the patient is relieved, the better the clinical outcome will be.^5^

In the 2010 American College of Rheumatology/European League Against rheumatism (ACR/EULAR) classification criteria,^6^ rheumatoid factor (RF) and anticitrullinated protein antibodies (ACPA) were recommended as a good marker for the diagnosis of RA. However, a percentage of patients with undifferentiated arthritis faces big challenges that arise from conventional diagnosis and classification criterion^7^ and RF is not very specific for RA and may also be detected in patients with other rheumatic disorders or infections, or in apparently healthy individuals.^8^ Therefore, it is necessary to incorporate some novel RA indicators into the diagnostic criteria of RA. 14‐3‐3 proteins are phospho‐serine/phospho‐threonine binding proteins able to associate with a wide range of protein targets, like kinases, phosphatases, transmembrane receptors and transcription factors.^9^ They are ubiquitously expressed in all eukaryotic organisms and by interacting with a multitude of functionally diverse and generally phosphorylated molecules, regulate a huge number of physiological processes, such as intracellular protein trafficking, cell proliferation, growth and apoptosis, regulation of metabolism, signal transduction and stress responses.^9^ 14‐3‐3 proteins are a group of highly conserved protein families, composed of 7 isoforms (β, γ, ε, η, σ, θ, and ζ).^9^ 14‐3‐3 η is 1 of 7 members of the 14‐3‐3 family that are preferentially expressed at higher concentrations in certain tissues, underscoring the importance of specific isoforms in the regulation of tissue‐specific functions.^10^ The association of 14‐3‐3 η protein and RA was reported for the first time in 2007 by Kilani et al^11^ .The research team found for the first time that serum 14‐3‐3 η protein was present at significantly higher levels in the synovial fluid and serum of patients with arthritis compared to healthy individuals and reported a positive association between 14‐3‐3 η and matrix metalloproteinases (MMPs).^11^ High level of 14‐3‐3 η was closely related to high expression of MMPs. Stimulating fibroblasts with recombinant 14‐3‐3 η, expression of MMP‐1 protein in fibroblasts was increased in a dose‐dependent fashion,^11^ and MMP‐1 and MMP‐9 were highly expressed after monocyte‐lineage THP‐1 cells were also treated with 14‐3‐3 η.^10^ MMPs have been confirmed to be responsible for the structure erosion of RA, as well as being a potential indicator of progressive joint damage and disease activity.^12^, ^13^, ^14^ These findings suggested that 14‐3‐3 η may be relevant to the course of cartilage and bone destruction, via regulation of the expression of MMPs. As a novel RA biomarker, serum 14‐3‐3 η protein levels are also associated to some extent with inflammatory responses and joint damage. Maksymowych et al designed an experiment in vitro to investigate the effect of 14‐3‐3 η on the activation of RA‐related signaling cascades and induction of proinflammatory mediators that contributed to RA joint damage.^10^ Cell stimulation studies revealed that 14‐3‐3 η preferentially stimulates cells of the innate immune system, leading to the activation of key signaling cascades such as MAPK/ERK, SAPK/JNK and the JAK‐STAT pathways that regulate the production of inflammatory factors such as interleukin (IL)‐1β, IL‐6; however, 14‐3‐3 η had no impact on p38MAPK phosphorylation.^10^ Moreover, 14‐3‐3 η caused the induction of factors directly correlated to the joint damage process, such as MMP‐1, MMP‐9 and receptor activator of nuclear factor kappa‐B ligand (RANKL) in a dose‐dependent manner.^10^ In addition, RA patients with 14‐3‐3 η‐positivity may have a higher incidence of osteoporosis. 14‐3‐3 η is thought to be involved in the development of osteoporosis in RA patients, and may be a predictor of osteoporosis in patients with early RA.^15^ Even if the diagnostic utility of serum 14‐3‐3 η protein as complementary biomarkers to RF/ACPA has been recently demonstrated in few studies,^16^, ^17^, ^18^ yet no systematic review or meta‐analysis has been conducted to assess its diagnostic value. Therefore, the aim of the present study was to summarize the published data on the sensitivity and specificity of 14‐3‐3 η protein and to evaluate its diagnostic performance for RA patients.

## 2 METHODS

### 2.1 Search strategy

We systematically searched the PubMed, EMBASE, and Web of Science, and studies published in English before 20 February 2020, that analyzed the diagnostic accuracy of 14‐3‐3 η protein were included. The following search terms were used: (“14‐3‐3 η” OR “14‐3‐3 eta” OR “14‐3‐3*”OR “YWHAH”) AND (“rheumatoid arthritis” OR "RA"). At the same time, all the references in the retrieved literatures were manually reviewed to identify other potential relevant articles. This meta‐analysis was performed based on the Preferred Reporting Items for Systematic Reviews and Meta Analyses (PRISMA) statement.^19^

### 2.2 Inclusion criteria

1. Diagnosis of RA conforms to 1987 ACR or 2010 ACR/ EULAR criteria^6^, ^20^ was regarded as the diagnostic gold standard.
2. Studies that enrolled healthy donors or patients without RA who have arthritis or other autoimmune diseases including osteoarthritis (OA), ankylosing spondylitis (AS), psoriatic arthritis (PsA), systemic lupus erythematosus (SLE), Sjögren's syndrome (SS), scleroderma (Scl), and dermatomyositis (DM).
3. Testing of 14‐3‐3 η protein by enzyme‐linked immunosorbent assay (ELISA).
4. All articles reporting either sensitivity or specificity of 14‐3‐3 η protein in RA or reporting data allowing calculation of sensitivity or specificity.
5. Samples in articles were restricted to human participants.
6. Accessible full text.

### 2.3 Exclusion criteria

1. Reviews, comments, conference abstracts, letters, editorials, and expert opinions.
2. Studies with insufficient data, specificity and sensitivity could not be obtained.
3. Specimen was not serum.
4. Non‐English articles.

### 2.4 Data extraction

The data were extracted by 2 investigators (Huiren Lei and Ting Yuan) independently. When a disagreement arose, the third investigator (Ding Cao) was consulted to confirm the extracted data. Author, year, gender ratio, mean age, ethnicity, case numbers, control groups, study design, and disease duration were extracted from each included study. Also, detection method, cut‐off value, diagnostic gold standard, true positive (TP) results, true negative (TN) results, false positive (FP) results, and false negative (FN) results were collected from each study.

### 2.5 Quality assessment

Quality of each study was assessed by 2 investigators (Tingguo Tang and Haiming Huang) independently using the Quality Assessment of Diagnostic Accuracy Studies 2 (QUADAS‐2) tool,^21^ which is specially developed to evaluate the quality of diagnostic tests. When disagreement occurred, the third investigator (Ding Cao) solved it. Four main domains indicated a set of signal questions contained in QUADAS‐2. It helps researchers reach the judgments regarding bias and applicability. Answers to each question should be “yes”, “no”, or “unclear”.

### 2.6 Statistical analysis

All data were analyzed using Stata 15, Meta‐Disc 1.4, and Rev Man 5.3. TP, FP, FN, and TN were used to calculate the sensitivity and specificity. A random effects model was produced to obtain combined sensitivity, specificity, diagnostic odds ratio (DOR), and positive/negative likelihood (LR+/LR−). Further, summary receiver operating characteristic was constructed to testify the summarized diagnostic rate. Cochran's *Q* test and *I* ^2^ statistic were applied to evaluate the heterogeneity.^22^ If heterogeneity existed (*P* < .05 or *I* ^2^ > 50%), subgroup analysis was performed to find out the reasons, and *I* ^2^ greater than 75% suggested there is substantial heterogeneity between studies.^23^ Deeks' funnel plot asymmetry test was used to evaluate the potential publication bias. *P* values less than .05 were considered significant difference.

## 3 RESULTS

**Figure 1:**
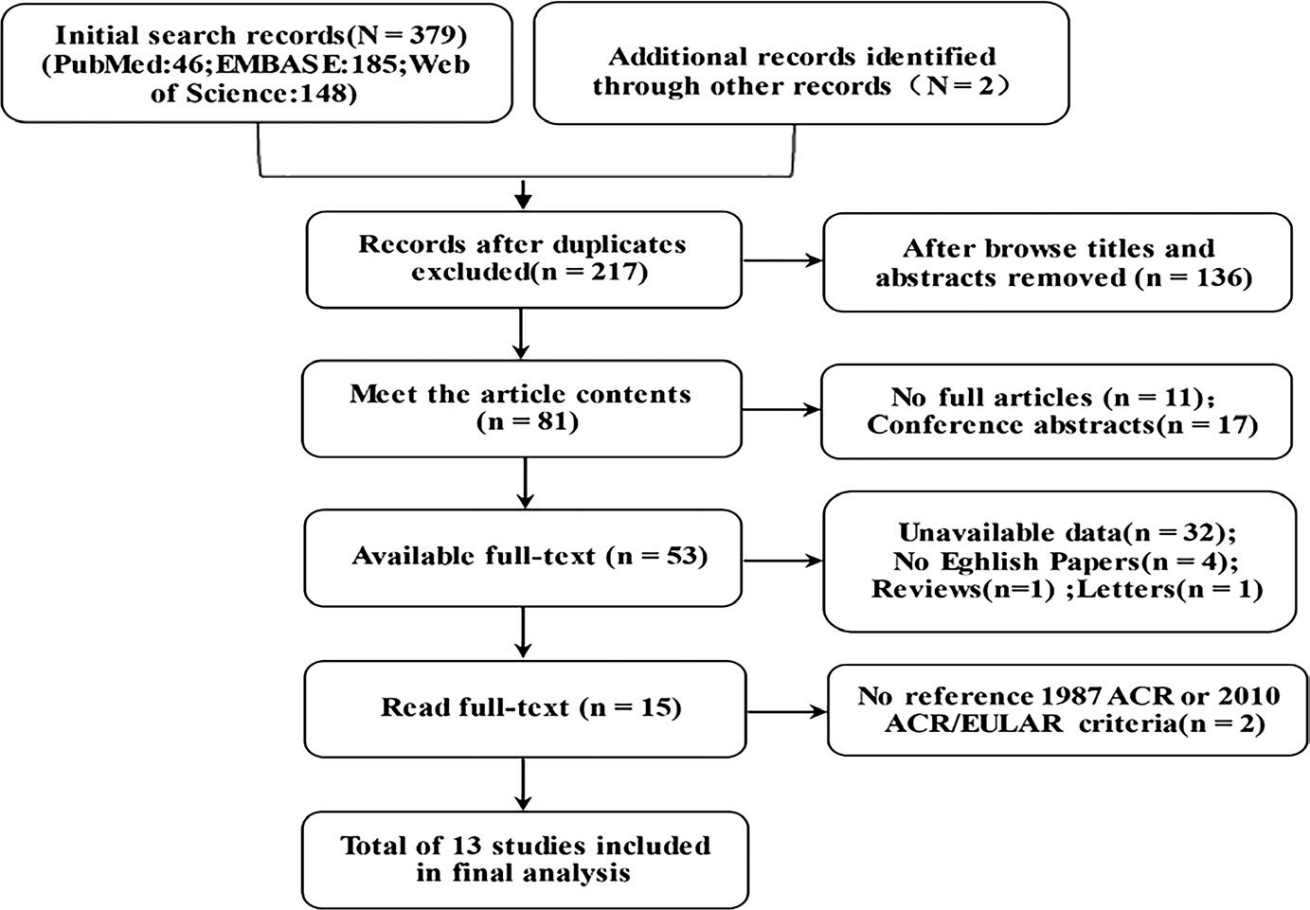
Flow diagram of screening studies

A total of 381 records were identified through databases (PubMed, EMBASE, and Web of Science) and by scrutinizing the reference lists of the included studies,^15^, ^16^, ^17^, ^18^, ^24^, ^25^, ^26^, ^27^, ^28^, ^29^, ^30^, ^31^, ^32^ of which 164 were excluded on account of duplicate records. Following thorough screening of the titles and abstracts, we excluded 136 of the remaining 217 studies due to no relevance to the topic or they were animal experiments. Only 81 articles remained for assessing full‐length papers. Of these articles, 11 failed to obtain full articles, 32 had lack of complete basic data, 17 were conference abstracts, 4 were not English papers, 1 was a letter, 1 was a review, and 2 did not meet the 1987 ACR/2010 ACR/EULAR criteria. Finally, 13 studies (1554 positive and 1934 negative participants) met the inclusion criteria and were included in this meta‐analysis. The study selection is shown on a flow diagram (Figure 1).

### 3.1 Studies' characteristics

The features of the 13 included articles are summed up in Tables 1 and 2. The publication date ranged from 2014 to 2020. Among the 13 studies, 7 were Asian populations, 3 were African populations, and 3 were European populations. The number of participants varied from 40 to 619. The mean age ranged from 42.0 to 61.0 years, and the female‐to‐male ratio in the included articles was from 0.25 to 29. In all studies, ELISA was used to detect 14‐3‐3 η protein. Additionally, the control groups were divided into 2 subgroups according to their different characteristics. Among these studies, both healthy people and other non‐RA rheumatic diseases were used as controls in 10 studies, while healthy people were used as controls in another 3 studies (Table 1).

**Table 1:** Some characteristics of the 13 studies included in the meta‐analysis of the diagnostic performance of the 14‐3‐3 η protein in rheumatoid arthritis

Author (Reference)	Year	Gender ratio	Mean age (y)	Ethnicity	Patient (control)	Control group	Study design	Disease duration (y)	SEN/SPE (ACPA)	SEN/SPE (RF)
Zeng^17^	2020	2.77	53.89	Asian	113 (289)	Disease control (212)	Case‐control	NR	76/96	75/80
						Healthy control (77)				
Huang^25^	2020	2.38	51.70	Asian	108 (192)	Disease control (90)	Case‐control	NR	72/94	77/79
						Healthy control (102)				
Salman^24^	2019	0.25	53.12	Asian	45 (45)	Healthy control (45)	Cross‐sectional	NR	NR	NR
Mohamed^18^	2017	8.20	44.32	African	92 (74)	Disease control (32)	Cohorts	eRA 1.17	NR	NR
						Healthy control (42)		Est. RA 7.06		
Guan^26^	2019	3.09	61.00	Asian	94 (80)	Disease control (40)	Cohorts	5	84/91	72/90
						Healthy control (40)				
Tan^28^	2018	2.94	42.00	Asian	128 (254)	Disease control (174)	Cohorts	6.10	80/97	NR
						Healthy control (80)				
Shovman^29^	2018	5.06	53.57	Asian	96 (167)	Disease control (101)	Cohorts	eRA < 1	NR	NR
						Healthy control (66)		Est. RA ≥ 1		
Gong^15^	2018	4.08	52.50	Asian	259 (80)	Healthy control (80)	Case‐control	eRA 0.8	NR	NR
								Est. RA 8		
Maksymowych^16^	2014	3.22	56.00	European	234 (385)	Disease control (196)	Cohorts	eRA 0.15	59/98	57/84
						Healthy control (189)		Est. RA 11.5		
Maksymowych^31^	2015	2.97	56.00	European	249 (251)	Disease control (196)	Cohorts	eRA 0.28	61/98	63/84
						Healthy control (55)		Est. RA 11.5		
Mohammed^27^	2019	19.00	45.85	African	20 (20)	Healthy control (20)	Case‐control	NR	65/100	85/100
Kadavath^32^	2014	3.14	50.00	European	91 (37)	Disease control (8)	Retrospective‐study	NR	NR	NR
						Healthy control (29)				
Elshahaly^30^	2018	29.00	45.50	African	30 (60)	Disease control (30)	Case‐control	NR	97/100	78/93
						Healthy control (30)				

Note

Gender ratio, female/male.

Abbreviations: ACPA, anti‐cyclic citrullinated peptide antibody; eRA, early RA; Est. RA, established RA; NR, not reported; RA, rheumatoid arthritis; RF, rheumatoid factor; SEN, sensitivity; SPE, specificity.

**Table 2:** The other characteristics of the 13 studies included in the meta‐analysis of the diagnostic performance of the 14‐3‐3 η protein in rheumatoid arthritis

Author (Reference)	Detection method	Cut‐off (ng/mL)	Criterion	TP	FP	FN	TN	SEN	SPE
Zeng^17^	ELISA	1.89	The 2010 ACR/EULAR criteria	82	23	31	266	73	92
Huang^25^	ELISA	2.60	The 2010 ACR/EULAR criteria	68	17	40	175	63	91
Salman^24^	ELISA	0.33	The 1987 ACR criteria OR 2010 ACR/EULAR criteria	40	8	5	37	89	82
Mohamed^18^	ELISA	0.39	The 2010 ACR/EULAR criteria	83	4	9	70	90	95
Guan^26^	ELISA	1.44	The 2010 ACR/EULAR criteria	74	21	20	59	79	74
Tan^28^	ELISA	NR	The 2010 ACR/EULAR criteria	66	19	62	235	52	93
Shovman^29^	ELISA	0.19	The 1987 ACR criteria OR 2010 ACR/EULAR criteria	48	8	48	159	50	95
Gong^15^	ELISA	0.88	The 2010 ACR/EULAR criteria	252	4	7	76	97	95
Maksymowych^16^	ELISA	0.19	The 1987 ACR criteria	167	54	67	331	71	86
Maksymowych^31^	ELISA	0.19	The 1987 ACR criteria	171	50	78	201	69	80
Mohammed^27^	ELISA	0.19	The 2010 ACR/EULAR criteria	18	2	2	18	90	90
Kadavath^32^	ELISA	0.20	The 2010 ACR/EULAR criteria	49	10	42	27	54	73
Elshahaly^30^	ELISA	0.20	The 2010 ACR/EULAR criteria	24	8	6	52	80	87

Abbreviations: 1987 ACR criteria: the American Rheumatism Association 1987 revised criteria for the classification of rheumatoid arthritis; 2010 ACR/EULAR criteria, the 2010 American College of Rheumatology/The European League Against Rheumatism classification criteria for RA; ELISA, enzyme‐linked immunosorbent assay; FN, false negative; FP, false positive; NR, not reported; SEN, sensitivity; SPE, specificity;TN, true negative; TP, true positive.

### 3.2 Study quality

The risks of bias and application concerns about reference standards in all included studies were low. The index test had almost 50% high risk of bias, while the application concerns of index test was low. About the domain of flow and timing in QUADAS‐2, the risks of bias in all included studies were low. Regarding the risks of bias about patient selection, all of the studies had high risk; however, the application concerns about patient selection in our meta‐analysis had high, unclear, and low, respectively (Figure S1).

### 3.3 Diagnostic accuracy of 14‐3‐3 η protein

**Figure 2:**
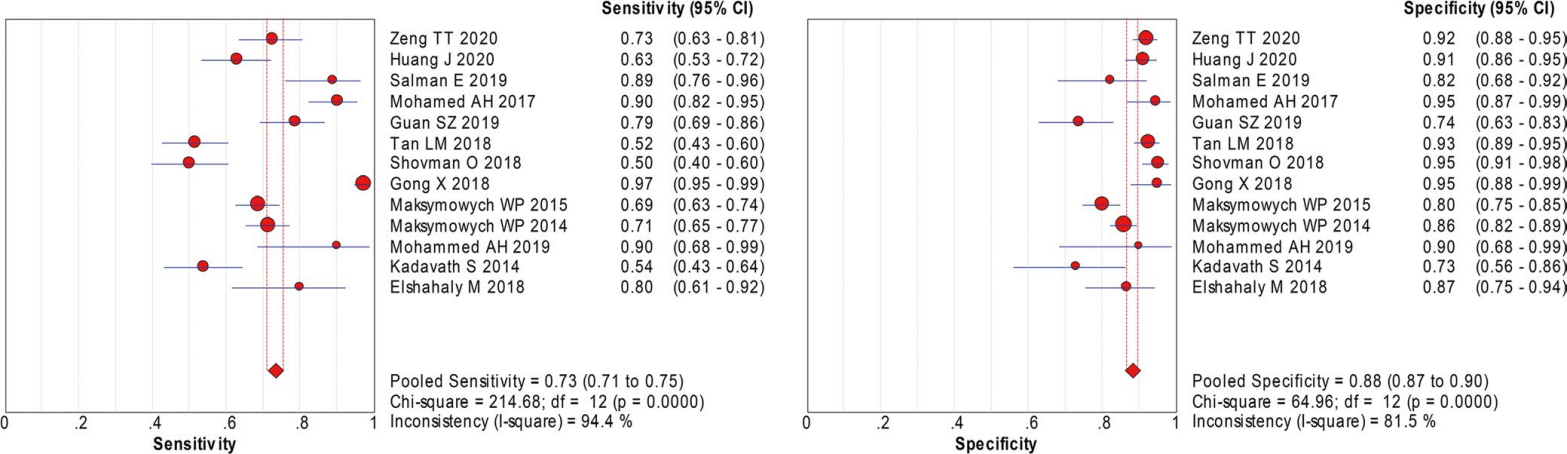
Forest plots for the diagnostic accuracy of each study (sensitivity and specificity)

**Figure 3:**
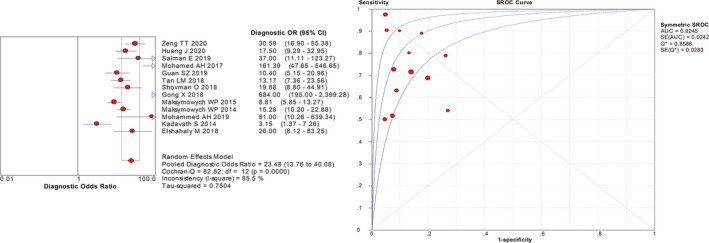
Forest plot of DOR and SROC curves of 14‐3‐3η protein in diagnosis of rheumatoid arthritis. DOR, diagnosis odds ratio; SROC, summary receiver operating characteristic

First, sensitivity, specificity, LR+, LR−, and DOR were counted to assess the diagnostic value for 14‐3‐3 η protein of RA. Since the *I* ^2^ of sensitivity and specificity was 94.4% and 81.5%, random effects model was applied to conclude the effective size. Then, the relevant pooled diagnostic values of 14‐3‐3 η protein were calculated. The pooled sensitivity and specificity were 0.73 (95% CI 0.71‐0.75) and 0.88 (95% CI 0.87‐0.90), respectively (Figure 2).The pooled LR + and LR − were 5.98 (95% CI 4.39‐8.14) and 0.28 (95% CI 0.21‐0.37), respectively (Figure S2). Additionally, the pooled DOR was 23.48 (95% CI 13.76‐40.08), and area under the curve (AUC) was 0.9245 (Figure 3).

### 3.4 Heterogeneity test

The heterogeneity of diagnostic tests included threshold effect and non‐threshold effect. The most important source of heterogeneity was due to different cut offs and threshold effect in all included studies.^33^ This analysis of diagnostic threshold conducted by Meta‐Disc 1.4 showed that logarithmic Spearman's correlation coefficient value of sensitivity (1‐specificity) was −0.077, with a *P* value of .803 (*P* > .5). This result indicated that this meta‐analysis did not have a threshold effect. Next, we carried out the subgroup analysis by Meta‐Disc 1.4 based on gender ratio, mean age, ethnicity, case number of RA patients, control groups, and study design. As shown in Table S1, after grouping, both the inconsistency (1‐square) of pooled sensitivity and specificity decreased in African and European groups, in Asian populations,^15^, ^17^, ^24^, ^25^, ^26^, ^28^, ^29^ the pooled sensitivity had no apparent change (0.73 [0.71‐0.75]) and the pooled specificity increased (0.91 [0.89‐0.93]); in African populations,^18^, ^27^, ^30^ both the pooled sensitivity (0.88 [0.82‐0.93]) and specificity (0.91 [0.85‐0.95]) augmented; however, in European groups,^16^, ^31^, ^32^ the pooled sensitivity (0.67 [0.63‐0.71]) and specificity (0.83 [0.80‐0.86]) declined. As shown in Table S1, in healthy and disease control,^16^, ^17^, ^18^, ^25^, ^26^, ^28^, ^29^, ^30^, ^31^, ^32^ the diagnostic value of pooled sensitivity decreased to 0.67 (0.65‐0.70) and pooled specificity had no apparent change (0.88 [0.86‐0.90]); however, in healthy controls,^15^, ^24^, ^27^ both the pooled sensitivity (0.96 [0.93‐0.98]) and specificity (0.90 [0.84‐0.95]) increased, and both the inconsistency (1‐square) of pooled sensitivity and specificity decreased. Overall, these results suggested that ethnicity and control groups may be a major source of heterogeneity. Finally, we performed sensitivity analysis to validate the stability of the meta‐analysis by consecutively omitting each of the enrolled studies. The results suggested no significant change using random‐effect methods when any 1 study was excluded. Sensitivity analysis indicated that the results of this meta‐analysis were stable (Figure S3).

### 3.5 Publication bias

**Figure 4:**
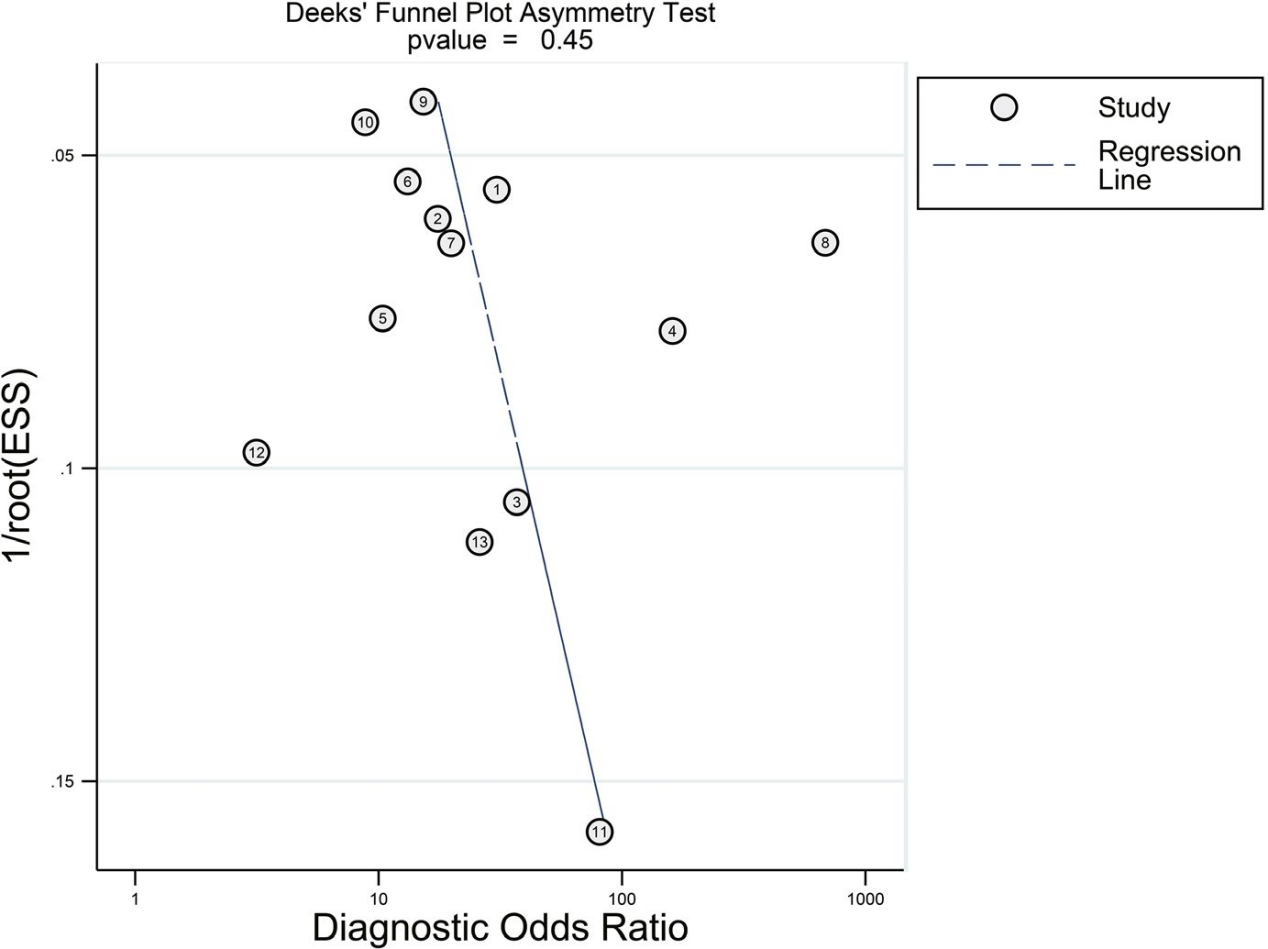
Funnel plots for detecting publications in this meta‐analysis

Deeks' Funnel Plot Asymmetry Test was performed to explore publication bias, and the *P* value was .45 (*P* > .05), which indicated there was little publication bias in this study (Figure 4).

## 4 DISCUSSION

Rheumatoid arthritis is 1 of the most prevalent chronic inflammatory diseases. It primarily involves the joints, but should be considered a syndrome that includes extra‐articular manifestations, such as rheumatoid nodules, pulmonary involvement or vasculitis, and systemic comorbidities.^34^ Early diagnosis combined with an accurate prognostic assessment is a core principle in the effective management of RA patients.^35^ At present, the specific pathogenesis of RA has not been clarified, but cytokines and inflammatory mediators are involved in the pathogenesis RA. 14‐3‐3 η is 1 of 7 members of the 14‐3‐3 family that is associated to some extent with inflammatory responses and joint damage in RA. Kilani et al^11^ found for the first time that serum 14‐3‐3 η protein was present at significantly higher levels in the synovial fluid and serum of patients with arthritis compared to healthy individuals and a positive association between 14‐3‐3 η and matrix metalloproteinase (MMPs) was reported, suggesting that 14‐3‐3 η may be relevant to the course of cartilage and bone destruction, via regulation of the expression of MMPs in patients with RA. To expand upon our understanding of the biological relevance of extracellular 14‐3‐3 η, Maksymowych et al performed in vitro cell signaling studies to determine if 14‐3‐3 η signals through the MAPK family, as well as through which family members. This family was selected because the transcription factor AP‐1, which resides downstream in the MAPK signaling nexus, is a key regulator of MMPs expression. The results indicate that stimulation of cells with 14‐3‐3 η, similar to tumor necrosis factor α (TNFα), results in the phosphorylation of both ERK and JNK/SAPK in a time‐dependent manner. However, unlike TNFα, 14‐3‐3 η had no impact on p38MAPK phosphorylation and that 14‐3‐3 η may play a role in perpetuating inflammation through the induction of factors such as IL‐6 and by exacerbating joint destruction via MMPs and RANKL. Examining 14‐3‐3 η′s expression in relation to clinical outcomes in RA patients will be of utmost importance in understanding how 14‐3‐3 η serum expression is used as a diagnostic test can assist clinicians with patient management. This meta‐analysis results provided evidence supporting 14‐3‐3 η protein as an auxiliary diagnostic serum marker in RA patients. A previous meta‐analysis found that the pooled sensitivity of ACPA and RF were 67% and 70%, respectively, and that pooled specificity of ACPA and RF were 96% and 86%, respectively.^36^ However, in this meta‐analysis, the pooled sensitivity of serum 14‐3‐3 η protein was 73%, which is much higher than RF (70%) and ACPA (67%), and the pooled specificity of serum 14‐3‐3 η protein was 88% that is higher than RF (86%) but is moderately lower than ACPA (96%). Although laboratory tests for the RA‐related autoantibodies ACPA is abnormal (seropositive) in many patients with RA, the value is normal (seronegative) in about one‐third of patients with RA.^37^ Seronegativity in cases of both early and established RA remains an important limitation of ACPA, emphasizing the need for new complementary markers to enhance diagnostic sensitivity. One of the advantages of 14‐3‐3η protein as an RA marker is that it can improve identification rates of seronegative RA (SNRA). Salman et al found that 40 (88%) of their 45 patients who were seronegative for RF and ACPA were 14‐3‐3η‐positive and suggested that 14‐3‐3η protein is a valuable and promising marker in patients with SNRA.^24^ Another of the advantages of 14‐3‐3 η protein as an RA marker is that adding 14‐3‐3 η to RF or ACPA or the combination of all 3 markers would increase the diagnostic rate. Guan et al found that adding 14‐3‐3 η to RF and ACPA testing increased diagnostic sensitivity for early RA patients.^26^ The results indicated that adding 14‐3‐3 η to ACPA and/or RF could discriminate more than 96% of patients with RA. The positive rate of at least 1 of the 3 markers was even up to 99%, with a specificity of about 70%. The utility of serum 14‐3‐3 η protein apparently elevating the detection rate for RA was consistent with the previous research results.^15^, ^16^, ^18^ Therefore, 14‐3‐3 η protein may contribute to the diagnosis of RA when combined with other antibodies detection and clinical manifestations. Our data also showed that the pooled LR + of 14‐3‐3 η protein was 5.98, which implied that the positive rate of 14‐3‐3 η protein in RA patients was 5.98 times than in non‐RA patients, and the LR– (0.28) was not low enough to exclude RA if 14‐3‐3 η protein test was negative. DOR, which ranges from 0 to infinity, reflects the links between the results of diagnostic tests and diseases; a higher value suggests that the diagnostic test has a stronger discriminatory ability between patients and healthy people. The study found that the pooled DOR was 23.48, and this result indicated 14‐3‐3 η protein was helpful in the diagnosis of RA. To demonstrate excellent accuracy, the value of AUC should be more than 0.97, and AUC of 0.75‐0.92 is considered to be good,^38^ whereas, AUC value was 0.9245 in this study which implied that 14‐3‐3 η protein had moderate diagnostic accuracy.

There was high heterogeneity among this meta‐analysis. Thus, we tried to analyze the source for heterogeneity. First, the definition of a positive or negative 14‐3‐3 η protein was defined with different cut‐off values; however, threshold effect was not found by Meta‐Disc 1.4. Second, we carried out the subgroup analysis based on gender ratio, mean age, ethnicity, case of RA patients, control groups, and study design (Table S1). We found that ethnicity and control groups may be major sources of heterogeneity. Additionally, some studies reported that the positive rate of 14‐3‐3 η protein in early RA (shorter than 1 year) was lower than that of the established RA (more than 1 year).^15^, ^16^, ^18^, ^31^ The studies in the meta‐analysis enrolled the RA patients with different disease durations, which may lead to heterogeneity as well. However, the information available in the included studies is too small to be analyzed, therefore, further studies are needed to expound the hypothesis.

This meta‐analysis has several limitations. First, some articles may have been missed which were not published in the databases we searched. Second, the current studies were mostly case‐control designs and most of the participants were clearly diagnosed. Therefore, well‐designed prospective studies with larger sample sizes are needed to confirm the diagnostic value of the 14‐3‐3 η protein for RA. Third, all patients enrolled in the trials are definite RA, with no difficult‐to‐diagnose patients included, which may lead to higher diagnostic evaluation. Fourth, a large heterogeneity in this meta‐analysis existed, so the random effects model and subgroup analysis were adopted to control the size of heterogeneity. In addition, the overall sample size of some studies was relatively small.

Nevertheless, this article is the first meta‐analysis describing the overall diagnostic value of 14‐3‐3 η protein in RA patients. Compared with the included individual studies, the major strength of this present study is more accurate results by synthesizing results from current published studies to evaluate the diagnostic value of 14‐3‐3 η protein in RA patients. Considering the shortcomings of this literature, relevant prospective experiments still need to be carried out.

## 5 CONCLUSION

From the results above, we confirmed that 14‐3‐3 η protein can be used as a complementary biomarker in the diagnosis of RA.

## CONFLICTS OF INTEREST

None.

## Supporting information

Figure S1

Figure S2

Figure S3

Table S1

## References

[1] Sparks JA . Rheumatoid arthritis. Ann Intern Med. 2019;170(1):ITC1‐ITC16. 30596879 10.7326/AITC201901010

[2] Smolen JS , Aletaha D , Barton A , et al. Rheumatoid arthritis. Nat Rev Dis Primers. 2018;4:18001 10.1038/nrdp.2018.1 29417936

[3] Aletaha D , Smolen JS . Diagnosis and management of rheumatoid arthritis: a review. JAMA. 2018;320(13):1360‐1372. 30285183 10.1001/jama.2018.13103

[4] Safiri S , Kolahi AA , Hoy D , et al. Global, regional and national burden of rheumatoid arthritis 1990–2017: a systematic analysis of the Global Burden of Disease study 2017. Ann Rheum Dis. 2019;78(11):1463‐1471. 31511227 10.1136/annrheumdis-2019-215920

[5] Akdemir G , Heimans L , Bergstra SA , et al. Clinical and radiological outcomes of 5‐year drug‐free remission‐steered treatment in patients with early arthritis: IMPROVED study. Ann Rheum Dis. 2018;77(1):111‐118. 28970207 10.1136/annrheumdis-2017-211375

[6] Aletaha D , Neogi T , Silman AJ , et al. 2010 rheumatoid arthritis classification criteria: an American College of Rheumatology/European League Against Rheumatism collaborative initiative. Ann Rheum Dis. 2010;69(9):1580‐1588. 20699241 10.1136/ard.2010.138461

[7] Brinkmann GH , Norli ES , Kvien TK , et al. Disease characteristics and rheumatoid arthritis development in patients with early undifferentiated arthritis: a 2‐year followup study. J Rheumatol. 2017;44(2):154‐161. 28089976 10.3899/jrheum.160693

[8] Egeland T , Munthe E . The role of the laboratory in rheumatology. Rheumatoid factors. Clin Rheum Dis. 1983;9(1):135‐160. 6347504

[9] Cau Y , Valensin D , Mori M , Draghi S , Botta M . Structure function, involvement in diseases and targeting of 14‐3‐3 proteins: an update. Curr Med Chem. 2018;25(1):5‐21. 28462702 10.2174/0929867324666170426095015

[10] Maksymowych WP , van der Heijde D , Allaart CF , et al. 14‐3‐3η is a novel mediator associated with the pathogenesis of rheumatoid arthritis and joint damage. Arthritis Res Ther. 2014;16(2):R99 10.1186/ar4547 24751211 PMC4060379

[11] Kilani RT , Maksymowych WP , Aitken A , et al. Detection of high levels of 2 specific isoforms of 14‐3‐3 proteins in synovial fluid from patients with joint inflammation. J Rheumatol. 2007;34(8):1650‐1657. 17611984

[12] Galil SM , El‐Shafey AM , Hagrass HA , Fawzy F , Sammak AEL . Baseline serum level of matrix metalloproteinase‐3 as a biomarker of progressive joint damage in rheumatoid arthritis patients. Int J Rheum Dis. 2016;19(4):377‐384. 25292349 10.1111/1756-185X.12434

[13] Ma JD , Wei XN , Zheng DH , et al. Continuously elevated serum matrix metalloproteinase‐3 for 3–6 months predict one‐year radiographic progression in rheumatoid arthritis: a prospective cohort study. Arthritis Res Ther. 2015;17:289 10.1186/s13075-015-0803-2 26467222 PMC4606896

[14] Green MJ , Gough AK , Devlin J , et al. Serum MMP‐3 and MMP‐1 and progression of joint damage in early rheumatoid arthritis. Rheumatology. 2003;42(1):83‐88. 12509618 10.1093/rheumatology/keg037

[15] Gong X , Xu SQ , Wu Y , et al. Elevated serum 14‐3‐3η protein may be helpful for diagnosis of early rheumatoid arthritis associated with secondary osteoporosis in Chinese population. Clin Rheumatol. 2017;36(11):2581‐2587. 28875246 10.1007/s10067-017-3807-2

[16] Maksymowych WP , Naides SJ , Bykerk V , et al. Serum 14‐3‐3η is a novel marker that complements current serological measurements to enhance detection of patients with rheumatoid arthritis. J Rheumatol. 2014;41(11):2104‐2113. 25128504 10.3899/jrheum.131446

[17] Zeng TT , Tan LM , Wu Y , et al. 14‐3‐3η protein in rheumatoid arthritis: promising diagnostic marker and independent risk factor for osteoporosis. Lab Med. 2020 lmaa001 . 10.1093/labmed/lmaa001 32080735

[18] Mohamed AH , Abdellatif S , El‐Noshokaty EH . Serum level of 14‐3‐3η (Eta) protein as a diagnostic marker for rheumatoid arthritis and potential correlation with disease activity. MOJ Orthop Rheumatol. 2017;7(4):280.

[19] Moher D , Liberati A , Tetzlaff J , Altman DG . Preferred reporting items for systematic reviews and meta‐analyses: the PRISMA statement. Int J Surg. 2010;8(5):336‐341. 20171303 10.1016/j.ijsu.2010.02.007

[20] Arnett FC , Edworthy SM , Bloch DA , et al. The American Rheumatism Association 1987 revised criteria for the classification of rheumatoid arthritis. Arthritis Rheum. 1988;31(3):315‐324. 3358796 10.1002/art.1780310302

[21] Whiting PF , Rutjes AW , Westwood ME , et al. QUADAS‐2: a revised tool for the quality assessment of diagnostic accuracy studies. Ann Intern Med. 2011;155(8):529‐536. 22007046 10.7326/0003-4819-155-8-201110180-00009

[22] Dinnes J , Deeks J , Kirby J , Roderick P . A methodological review of how heterogeneity has been examined in systematic reviews of diagnostic test accuracy. Health Technol Assess. 2005;9(12):1‐113. 10.3310/hta9120 15774235

[23] Higgins JP , Thompson SG , Deeks JJ , Altman DG . Measuring inconsistency in meta‐analyses. BMJ. 2003;327(7414):557‐560. 12958120 10.1136/bmj.327.7414.557 PMC192859

[24] Salman E , Çetiner S , Boral B , et al. Importance of 14‐3‐3eta, anti‐CarP, and anti‐Sa in the diagnosis of seronegative rheumatoid arthritis.*Turk* . J Med Sci. 2019;49(5):1498‐1502. 10.3906/sag-1812-137 PMC7018368 31651120

[25] Huang J , Zeng T , Zhang X , et al. Clinical diagnostic significance of 14‐3‐3η protein, high‐mobility group box‐1, anti‐cyclic citrullinated peptide antibodies, anti‐mutated citrullinated vimentin antibodies and rheumatoid factor in rheumatoid arthritis. Brit J Biomed Sci. 2020;77(1):19‐23. 31433746 10.1080/09674845.2019.1658425

[26] Guan SZ , Yang YQ , Bai X , et al. Serum 14‐3‐3η could improve the diagnostic rate of rheumatoid arthritis and correlates to disease activity. Ann Clin Lab Sci. 2019;49(1):57‐62. 30814078

[27] Mohammed AH , Rasha MG , Wagenat EA , et al. Assessment Of 14‐3‐3 protein eta And Anti‐Cyclic Citrullinated Peptide (ANTI‐CCP) in juvenile idiopathic arthritis versus adult onset rheumatoid arthritis. Al Azhar Med J. 2019;48(3):243‐256.

[28] Tan LM , Wang QH , Zeng TT , et al. Clinical significance of detecting HLA‐DR, 14‐3‐3η protein and d‐dimer in the diagnosis of rheumatoid arthritis. Biomarkers Med. 2018;12(7):697‐705. 10.2217/bmm-2017-0371 29856230

[29] Shovman O , Gilburd B , Watad A , et al. The diagnostic value of 14‐3‐3η protein levels in patients with rheumatoid arthritis. Best Pract Res Clin Rheumatol. 2018;32(4):610‐617. 31174829 10.1016/j.berh.2019.01.010

[30] Elshahaly M , Saleh M , Fahmy H , Othman M . AB0273 Assessment of serum levels of 14–3–3η protein in rheumatoid arthritis: is it a specific marker for the disease? Ann Rheum Dis. 2018;77(Suppl 2):1316.

[31] Maksymowych WP , Boire G , van Schaardenburg D , et al. 14‐3‐3η autoantibodies: diagnostic use in early rheumatoid arthritis. J Rheumatol. 2015;42(9):1587‐1594. 26178283 10.3899/jrheum.141385

[32] Kadavath S , Chittalae S , Shuaib ON , et al. SAT0211 14‐3‐3 ETA protein: a novel biomarker for the diagnosis of rheumatoid arthritis. Ann Rheum Dis. 2014;73(Suppl 2):666.

[33] Besada E , Nikolaisen C , Nossent H . Diagnostic value of antibodies against mutated citrullinated vimentin for rheumatoid arthritis. Clin Exp Rheumatol. 2011;29(1):85‐88. 21269572

[34] Smolen JS , Aletaha D , McInnes IB . Rheumatoid arthritis. Lancet. 2016;388(10055):2023‐2038. 27156434 10.1016/S0140-6736(16)30173-8

[35] Littlejohn EA , Monrad SU . Early diagnosis and treatment of rheumatoid arthritis. Prim Care. 2018;45(2):237‐255. 29759122 10.1016/j.pop.2018.02.010

[36] Whiting PF , Smidt N , Sterne JA , et al. Systematic review: accuracy of anti‐citrullinated Peptide antibodies for diagnosing rheumatoid arthritis. Ann Intern Med. 2010;152(7):456‐464; W155–66. 20368651 10.7326/0003-4819-152-7-201004060-00010

[37] Avouac J , Gossec L , Dougados M . Diagnostic and predictive value of anti‐cyclic citrullinated protein antibodies in rheumatoid arthritis: a systematic literature review. Ann Rheum Dis. 2006;65(7):845‐851. 16606649 10.1136/ard.2006.051391 PMC1798205

[38] Walter SD . Properties of the summary receiver operating characteristic (SROC) curve for diagnostic test data. Stat Med. 2002;21(9):1237‐1256. 12111876 10.1002/sim.1099

