# Supplementary figures and images for: Diagnostic accuracy of 14‐3‐3 η protein in rheumatoid arthritis: A meta‐analysis
Source: Int J Rheum Dis. 2020 Sep 10;23(11):1443–51. doi: 10.1111/1756-185X.13921 (PMC7756802; doi:10.1111/1756-185X.13921)

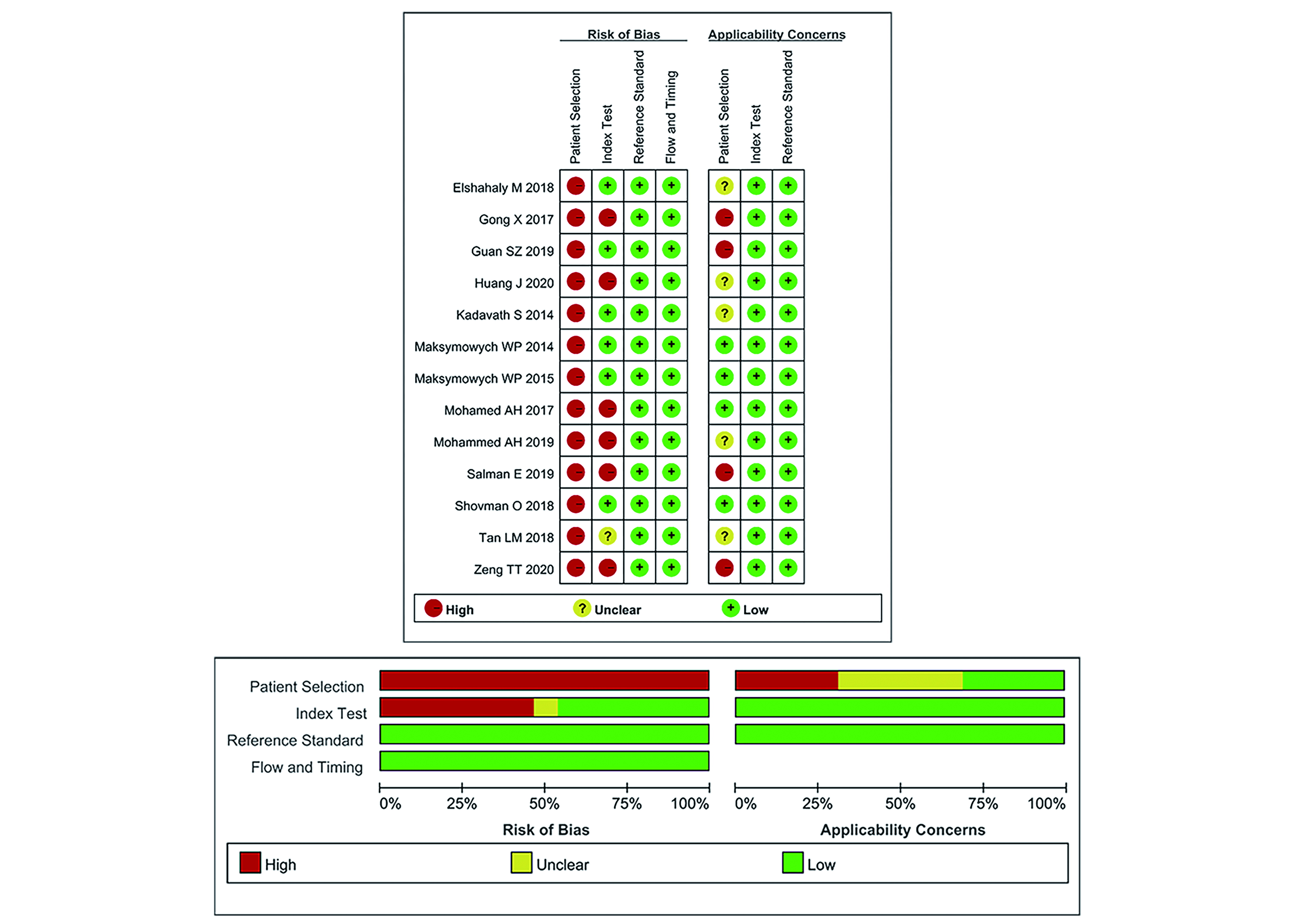

Supplement: Supplementary file 1 — Figure S1 [file APL-23-1443-s001.tif]

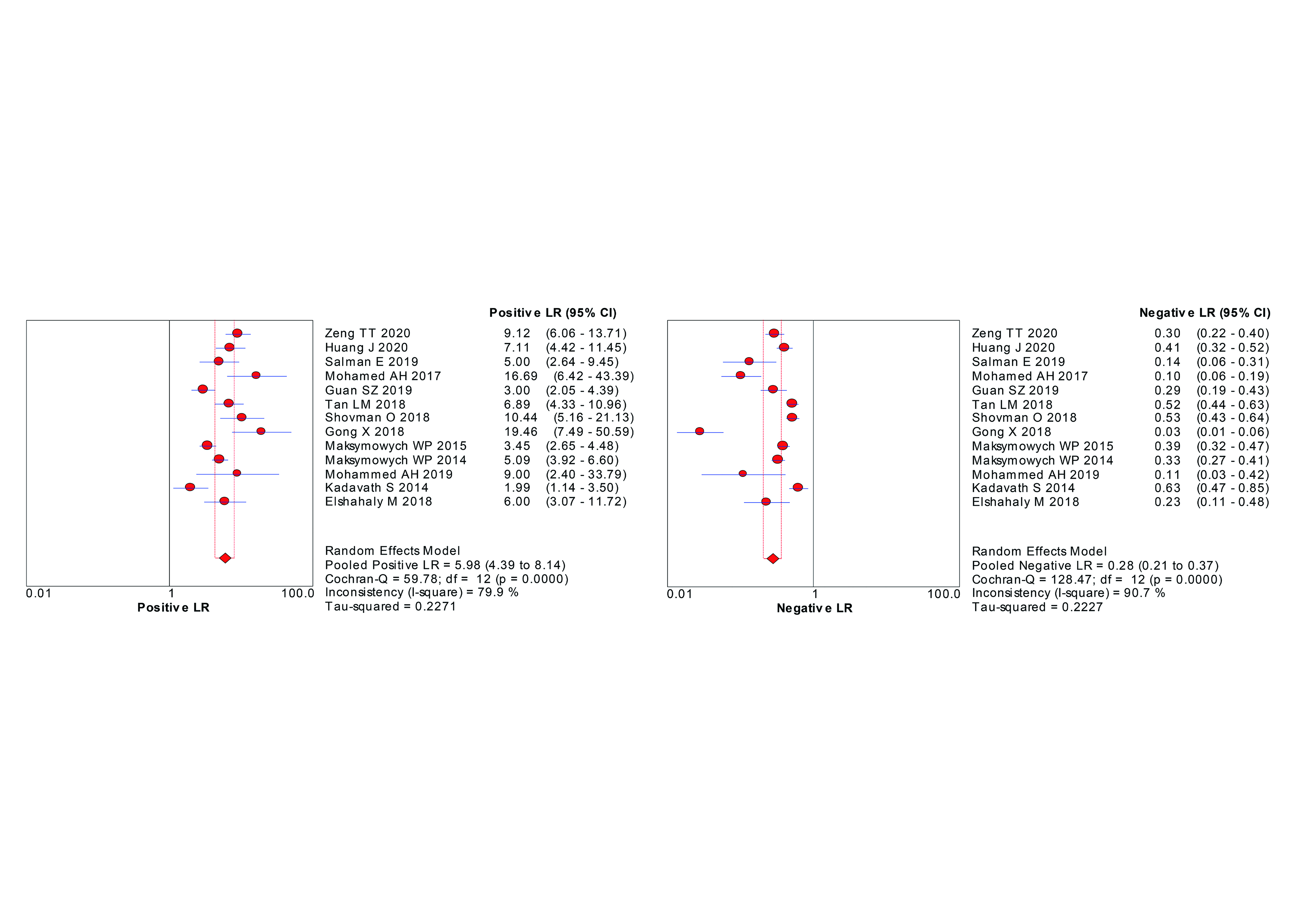

Supplement: Supplementary file 2 — Figure S2 [file APL-23-1443-s002.tif]

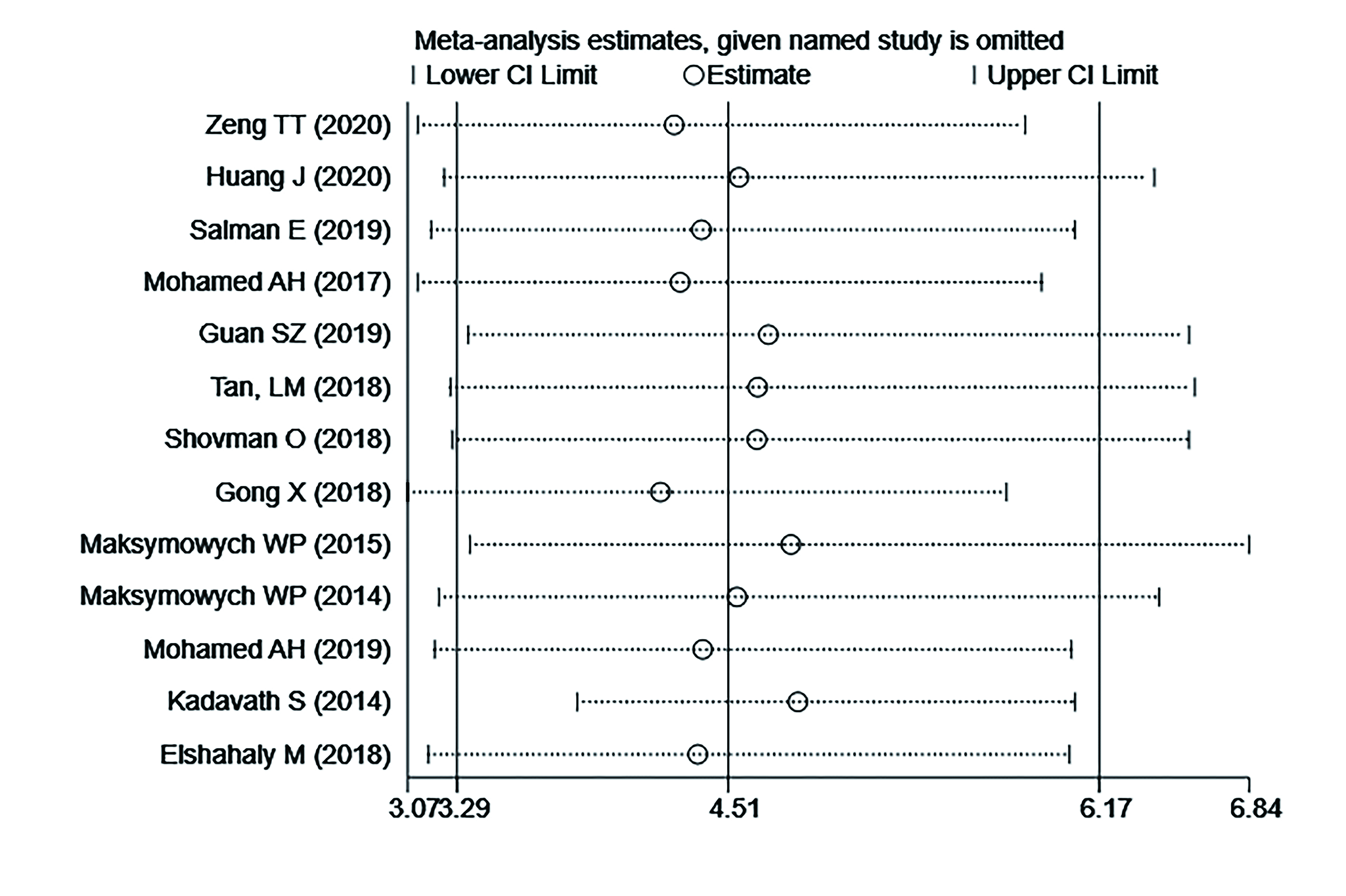

Supplement: Supplementary file 3 — Figure S3 [file APL-23-1443-s003.tif]
