# Supplementary material for: Diagnostic accuracy of 14‐3‐3 η protein in rheumatoid arthritis: A meta‐analysis
Source: Int J Rheum Dis. 2020 Sep 10;23(11):1443–51. doi: 10.1111/1756-185X.13921 (PMC7756802; doi:10.1111/1756-185X.13921)
Supplement: Supplementary file 4 — Table S1 [file APL-23-1443-s004.docx]

**Supplementary Table 3. Subgroup analysis of the diagnostic performance of the 14-3-3 η protein in rheumatoid arthritis**

| **Subgroup** | **No. of studies** | | **SEN(95% CI)** | **SPE(95% CI)** | **Heterogeneity test** | | |
| --- | --- | --- | --- | --- | --- | --- | --- |
|  |  |  | |  | ***Q*(SEN/SPE)** | ***I²* (%)(SEN/SPN)** | ****P*(SEN/SPN)** |
| *Total* | 13 | 0.73( 0.71-0.75) | | 0.88 (0.87-0.90) | 214.68/64.96 | 94.4/81.5 | 0.0000/0.0000 |
| *Ethnics* |  |  | |  |  |  |  |
| Asian  **African** | 7  **3** | 0.73( 0.71-0.75)  **0.88( 0.82-0.93)** | | 0.91 (0.89-0.93)  **0.91( 0.85-0.95)** | 214.68/30.83  **2.09/2.58** | 94.4/80.5  **4.2/22.5** | 0.0000/0.0000  **0.3520/0.2750** |
| European  *Control groups* | 3 | 0.67( 0.63-0.71) | | 0.83( 0.80-0.86) | 9.10/6.31 | 78.0/68.3 | 0.0106/0.0427 |
| **Healthy control**  Healthy+ disease control  *Sex ratio*  ≥2  ＜2  *Mean age*  ≥50  ＜50  *Case numbers*  ≥100  ＜100  *Study design*  Case-control | **3**  10  12  1  9  4  6  7  5 | **0.96 (0.93-0.98)**  0.67 (0.65-0.70)  0.73(0.70-0.75)  NC  0.74(0.71-0.76)  0.71(0.65-0.76)  0.74(0.71-0.76)  0.72(0.67-0.76)  0.84(0.80-0.87) | | **0.90 (0.84-0.95)**  0.88(0.86-0.90)  0.88(0.87-0.90)  NC  0.87(0.85-0.89)  0.92(0.89-0.94)  0.88(0.87-0.90)  0.87(0.84-0.90)  0.92(0.89-0.94) | **6.59/5.17**  72.61/59.07  207.83/63.56  NC  166.52/54.69  47.13/2.91  146.52/31.03  67.45/33.50  87.46/3.21 | **69.7/61.3**  87.6/84.8  94.7/82.7  NC  95.2/85.4  93.6/0  96.6/83.9  91.1/82.1  95.4/0 | **0.0370/0.0754**  0.0000/0.0000  0.0000/0.0000  NC  0.0000/0.0000  0.0000/0.4054  0.0000/0.0000  0.0000/0.0000  0.0000/0.5230 |
| No case control | 8 | 0.68(0.65-0.71) | | 0.87(0.85-0.88) | 79.18/50.77 | 91.2/86.2 | 0.0000/0.0000 |

Sex ratio: female/male; SEN: sensitivity; SPE: specificity; CI: confidence interval; *Q* (SEN/SPE): the pooled sensitivity of Chi-square /the pooled specificity of Chi-square; *I²* (SEN/SPE): the pooled sensitivity of Inconsistency (1-square) /the pooled specificity of Inconsistency (1-square); NC: not calculated; **p*-value for heterogeneity based on *Q* (SEN/SPE) test; Significant results are marked in bold.
